# Multispectral images of flowers reveal the adaptive significance of using long-wavelength-sensitive receptors for edge detection in bees

**DOI:** 10.1007/s00359-017-1156-x

**Published:** 2017-03-17

**Authors:** Vera Vasas, Daniel Hanley, Peter G. Kevan, Lars Chittka

**Affiliations:** 1 Bee Sensory and Behavioural Ecology Lab, School of Biological and Chemical Sciences, Queen Mary University of London, London, UK; 2 Department of Biology, Long Island University - Post, Brookville, NY USA; 3 School of Environmental Sciences, University of Guelph, Guelph, ON N3C 2W1 Canada

**Keywords:** Color processing, Receptor adaptation, Signal-to-noise ratio, Visual ecology, Visual processing

## Abstract

Many pollinating insects acquire their entire nutrition from visiting flowers, and they must therefore be efficient both at detecting flowers and at recognizing familiar rewarding flower types. A crucial first step in recognition is the identification of edges and the segmentation of the visual field into areas that belong together. Honeybees and bumblebees acquire visual information through three types of photoreceptors; however, they only use a single receptor type—the one sensitive to longer wavelengths—for edge detection and movement detection. Here, we show that these long-wavelength receptors (peak sensitivity at ~544 nm, i.e., green) provide the most consistent signals in response to natural objects. Using our multispectral image database of flowering plants, we found that long-wavelength receptor responses had, depending on the specific scenario, up to four times higher signal-to-noise ratios than the short- and medium-wavelength receptors. The reliability of the long-wavelength receptors emerges from an intricate interaction between flower coloration and the bee’s visual system. This finding highlights the adaptive significance of bees using only long-wavelength receptors to locate flowers among leaves, before using information provided by all three receptors to distinguish the rewarding flower species through trichromatic color vision.

**Electronic supplementary material:**

The online version of this article (doi:10.1007/s00359-017-1156-x) contains supplementary material, which is available to authorized users.

## Introduction

Most species of bees, like humans, have trichromatic color vision and excellent color discrimination (Kevan and Backhaus 1998; Chittka and Wells 2004; Dyer and Neumeyer 2005). They acquire visual information through three types of photoreceptors, with peak sensitivities in the short- (S, ${{\lambda }_{\max }}$ ≈ 344 nm), medium- (M, ${{\lambda }_{\max }}$ ≈ 436 nm) and long-wavelength (L, ${{\lambda }_{\max }}$ ≈ 544 nm) regions of their visual spectrum (Menzel and Blakers 1976; Peitsch et al. 1992) (Supplementary Material, Fig. S1). However, they appear to use the inputs to the long-wavelength receptors exclusively for a number of tasks. Long-wavelength contrast guides landing behavior (Lehrer et al. 1990; note that landing often takes place on a moving target; Mirwan and Kevan 2015), and long-wavelength inputs are used in learning the orientation of edges (Giger and Srinivasan 1996), in motion detection (Kaiser 1975; Lehrer et al. 1988) and in other visual tasks relying on motion (Srinivasan et al. 1989; Spaethe et al. 2001; Chittka and Tautz 2003). Moreover, spatial acuity also plays a role in the choice of receptor type. Target detection at the limit of the honeybee’s eye’s resolution (where the visual angle subtended by the stimuli is less than 15°) makes use only of long-wavelength inputs (Giurfa et al. 1996; Dyer et al. 2008). Long-wavelength receptors also respond faster to stimulus changes in bumblebees (Skorupski and Chittka 2010, 2011).

Indeed in other animals with excellent color vision, the detection of edges, movement and flicker remains achromatic (Jacobs 2013). Motion detection is largely achromatic because including color information would compromise the sensitivity to movement (Srinivasan 1985); for ideal movement detection, two receptors, located at different places in the eye, must give the very same signal to the same object when it is moving across the retina. In the case of edge detection and other tasks, achromatic coding may be advantageous through enhanced speed and efficiency of processing; however, whatever the reason, discarding color information for multiple visual tasks appears to be the rule rather than the exception. In mammals, edge detection and movement detection depend on a single class of cone, which has its peak sensitivity in the range 510–570 nm (Jacobs 2013). Humans, in particular, employ the responses of two long-wavelength receptors (green and red, peaking at 540 and 570 nm, respectively) to deal with the detection of edges and spatial detail (Lee et al. 2012), while blue receptors (${{\lambda }_{\max }}$ ≈ 430 nm) have little role in this process (Mollon 1989). In insects, the visual systems of particular species appear to be variations on one theme, and in this theme short visual fibers with long-wavelength (green) sensitivity provide the input to movement detectors (reviews: Kaiser 1975; Briscoe and Chittka 2001, flies; Takemura et al. 2013, locusts; Osorio 1986).

Edge detection, orientation detection and motion detection (in the context of active vision; Egelhaaf et al. 2012) are all facets of the daily task of navigating and finding salient cues in a complex heterogeneous environment. A prominent visual task of foraging bees is to locate, recognize and discriminate between reward-producing flowers. Due to the mutualistic nature of plant–pollinator interactions, bee-pollinated flowers are expected to have evolved displays aimed at bees (Sprengel 1793; Darwin 1888; Menzel and Shmida 1993; Kevan and Backhaus 1998; Gumbert et al. 1999), and bees are expected to have a visual system that is efficient at detecting bee-pollinated flowers (Chittka and Menzel 1992; Chittka et al. 1993; Kevan and Backhaus 1998). We address the advantage of using only the long-wavelength channel for so many tasks within this adaptive framework.

We propose that the usefulness of long-wavelength receptors might lie in the signal-to-noise ratio of their responses. In noisy surroundings, some receptor types provide more consistent signals in response to natural objects. Segregation of the visual field into elements that belong together is an important preliminary to the actual recognition of objects (Mollon 1989), and correct group assignment is easier when the objects elicit more intense and less variable receptor responses. Addressing this hypothesis requires examining the bees’ color perception across the complex spatial structure of the flowers themselves. Therefore, we have compiled a floral color database based on black-and-white photographs that used a series of broadband-pass optical filters that transmit across the visual range of bees (using the methodology described in Kevan 1972 and improved upon with more sophisticated equipment as described in Chittka and Kevan 2005) and employed custom-made computer programs to generate multispectral images. The multispectral images enabled us to model the neuronal responses that the bees’ photoreceptors produce in response to these floral stimuli, allowing us to compare the role of the different spectral channels in color processing. In particular, we estimated (1) the variation in the responses of the three different receptor types across a variety of flower species and (2) the signal content of each individual flower’s petals and leaves. Our results offer an adaptive explanation for choosing the long-wavelength channel over shorter-wavelength channels in the first steps of interpreting a visual scene and point out a striking example for the ways ecology and early perception can shape more downstream neural processes.

## Methods

### The flower image database

**Fig. 1:**
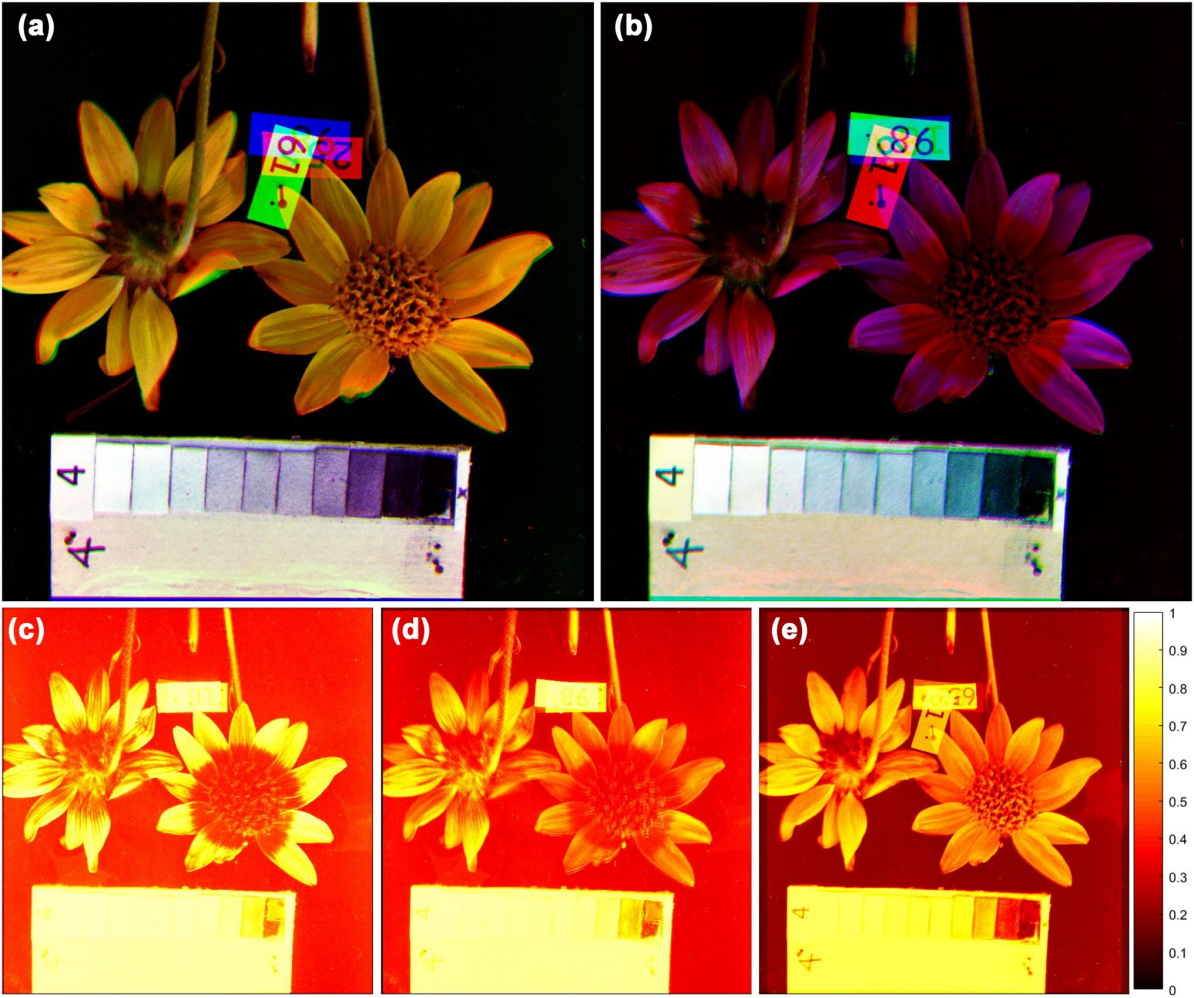
Example of the image analysis (*Arnica mollis*). *Black*-and-*white* photographs were taken through a series of monochromatic filters (Kevan 1972) with peak transmissions at 340, 400, 460, 520, 580, 640 and 700 nm. The photographs have been corrected for *white* balance based on *gray scales*. We tinted the *black*-and-*white* images to the appropriate hue and combined them to result in a false color image. For human vision (**a**), we combined the *blue*, *green* and *red layers*; for bees (**b**), we transformed *yellow*, *blue* and ultraviolet (invisible to human observers), into *red*, *green* and *blue*. We then calculated the dimensionless receptor responses of the short- (**c**), medium- (**d**) and long-wavelength receptors (**e**) that describe the electrical signals the bees’ photoreceptors would produce in response to the image. Response values are represented by the brightness of each pixel in the heat maps

The multispectral images (Fig. 1) are available to download from the Floral Image Database (http://liu.edu/flower). Black-and-white photographs were taken through a series of monochromatic filters depicting flowers from the alpine vegetation of Pennsylvania Mountain in the Rocky Mountains near Fairplay, Colorado, during the summers of 1978–1981 (following the methodology described in Kevan 1972; Chittka and Kevan 2005). The band-pass filters had broadband transmissions and collectively covered the full wavelength range of bee vision (peak transmissions at 340, 400, 460, 520, 580, 640 and 700 nm; the importance of covering the full visual spectrum is discussed in Kevan 1979; Kevan et al. 2001). Most importantly, the photographs include a UV-reflecting grayscale (Kevan et al. 1973; Kevan 1979; Chittka and Kevan 2005) that served as the check for correct exposures and then as reference point to which we adjusted the white balance of each photograph. There are 53 images (of 49 species) in the database (Supplementary Material, Table S1).

These images were scanned using a Canon LIDE 210 scanner and processed in ImageJ (Rasband 1997). Photographs taken of the same flower but through different filters were organized into stacks with seven layers. White balance was adjusted based on the grayscale for each layer, setting the darkest pixel black and lightest white with the java plug-in Color Correct (http://www.mecourse.com/landinig/software/software.html, for further details see Supplementary Data, Hanley et al. 2013). Another plug-in aligned the separate images of the same flowers with a normalized cross-correlation method (https://sites.google.com/site/qingzongtseng/template-matching-ij-plugin).

When comparing the reflectance spectra of different flower parts, we manually selected areas on the photographs that depicted the display structures (petals), the areas where the nectar and the stamens/carpels can be found (centers) and leaves. The cropped images were realigned (by the plug-in mentioned above) in order to achieve the best overlap between layers. A total of 52 photographs included petals, 38 photographs included centers and 35 photographs included leaves (Supplementary Material, Table S1). From each area, we randomly selected 1000 pixels and calculated their mean and standard deviation. We used these estimations for describing the average spectral reflectance functions of the petals, centers and leaves of the 49 plant species (1000 pixels were sufficient to represent the spectra, see Supplementary Material, Fig. S2).

In addition to providing spatially explicit reflectance measurements, we wished to supply the readers with images to promote the intuitive understanding of color and pattern, both as humans and as bees see them. Therefore, we included false-colored images in the database. We used ImageJ (Rasband 1997) to tint the black-and-white images to the hue defined by the wavelength of the filter that was used to take them and combined them to result in a normal (RGB) color image. For human vision, the combination of blue, green and red layers reproduces the original color reasonably well. For bees, we selected the UV, blue and yellow layers and tinted them blue, green and red to produce false color images (i.e., shifted the color so that UV becomes blue, blue becomes green, and green becomes red, as per the paradigm used by Kevan 1972, 1978; Mulligan and Kevan 1973; Fig. 1).

### Calculating receptor responses

Our understanding of the phototransduction process is detailed enough to predict the electrical signals the bees’ photoreceptors will produce when viewing a particular object (Backhaus and Menzel 1987; Chittka and Kevan 2005). The relative amount of light (quantum catch) absorbed by a particular type of photoreceptor can be calculated as:

1 $$P=R\int\limits_{300}^{700}{{{I}_{\text{s}}}}(\lambda )S(\lambda )D(\lambda )\text{d}\lambda \text{,}$$

where ${{I}_{\text{s}}}(\lambda )$ is the spectral reflectance function of the stimulus, $S(\lambda )$ is the spectral sensitivity function of the receptor, and $D(\lambda )$ is the spectrum of the illuminant (Naka and Rushton 1966; Laughlin 1981). The parameter *R* is called the sensitivity factor, and it is the result of the receptors’ tendency to adapt to a given ambient light level or background stimulus: over extended periods receptors’ sensitivities increase when they are poorly stimulated and decrease when they are strongly stimulated. *R* is calculated as:

2 $$R=1/\int\limits_{300}^{700}{{{I}_{\text{B}}}}(\lambda )S(\lambda )D(\lambda )\text{d}\lambda \text{,}$$

where ${{I}_{\text{B}}}(\lambda )$ is the spectral reflectance function of the stimulus the receptor is adapted to (i.e., the background; Laughlin 1981). This is in line with the von Kries adaptation theory (von Kries 1905), which is based on the assumption that the sensitivity of a photoreceptor is scaled depending on the overall intensity of the light in the receptor’s spectral domain.

The normalized (and so dimensionless) receptor response, *E*, is directly calculated from the quantum catch *P* (Backhaus and Menzel 1987, simplified from; Naka and Rushton 1966):

3 $$E=P/(P+1)$$

$E$can obtain values between 0 (baseline response) and 1 (maximal response) and is assumed to display half-maximal response (*E* = 0.5) to the light reflected from the adaptation background. The shape of the response curve provides a near-logarithmic coding that enables the visual system to calculate light intensity ratios with simple addition and subtraction (ratios of quantum catches, not absolute differences, are useful for comparing the reflectance properties of objects).

We used Python to implement these calculations (the script is available to download from www.insectvision.org). By default, the program uses the photoreceptor sensitivities of the honeybee from Peitsch et al. (1992), the standard daylight illumination function D65 (Wyszecki and Stiles 1982) and the reflectance spectrum of a typical green leaf (Chittka et al. 1994) as a background (for the functions, see Supplementary Material, Fig. S3). The calculations in the program are discretized and can be run at any resolution. We have seven data points from each reflectance function across the 300–700 nm range of the spectrum (one corresponding to each filter available); therefore, photoreceptor spectral sensitivity functions and the daylight illumination functions were used at the same low spectral resolution (i.e., a data point every 60 nm). We made sure to keep the total sensitivities of the receptors (the summed up sensitivity across all wavelengths) constant relative to each other (Supplementary Material, Table S2). We verified that this low-resolution model is sufficient to estimate the receptor responses accurately by downloading high-resolution flower reflectance curves from the Flower Reflectance Database (Arnold et al. 2010), lowering their resolution to match those used in our model and comparing the receptor responses calculated from the high- and low-resolution data (Supplementary Material, Fig. S4). We set the spectral reflectance function of the background to the mean of the 35 leaves that were included in the photographs, assuming that they represent the typical green foliage in the particular habitat (Supplementary Material, Fig. S3).

From each of the 52 petals, 38 centers and 35 leaves, we used 1000 pixels to estimate the short-, medium- and long-wavelength receptor responses. First, we calculated the receptor responses to the average reflectance function of the chosen area, with the aim of describing flower and leaf colors in general as bees see them. Second, we estimated the receptor responses to each pixel and calculated their mean, standard deviation and the signal-to-noise ratio (SNR, defined here as mean/standard deviation) of each area. We compared the receptor responses and the SNRs across the different types of receptors.

Next, we addressed the effects the specifics of the bee eye—the sensitivity functions of the receptors and the way they adapt to the illumination and to the color of the background—and the flower petal colors have on the receptor responses. With this aim, we constructed a null model, using unit background reflectance, unit illumination and unit receptor sensitivities in the calculations (see Supplementary Material, Table S3), and run it on 10,000 spectra with randomly generated reflectance intensities. We then calculated the mean and the standard deviations of quantum catches (*P*) and relative receptor responses (*E*) as a function of the peak sensitivities of the receptors. Finally, we assessed the effects of illumination spectrum, background reflectance spectrum, the shape of the receptor’s sensitivity function, and realistic reflectance functions by adding all combinations of these factors to the null model.

With the aim of promoting an intuitive, visual understanding of our conclusions, we also included in our dataset heat maps that depict the short-, medium- and long-wavelength receptor responses to each multispectral image (for an example, see Fig. 1b; the heat maps are available to download from http://liu.edu/flower). Two types of heat maps are provided, (1) for each of the 53 photographs and (2) for five examples where, from the same photograph, petals were manually cropped and pasted on top of leaves. We chose these five species because their leaves’ reflectance was close to the ‘average leaf reflectance,’ i.e., they could be used to represent the typical background. It is important to note here that the compound eye of the honeybee contains approximately 5000 ommatidia, each receiving light from only one small fragment of the visual field (Jander and Jander 2002); therefore, the spatial resolution of the two bee eyes is fairly low. For this reason, we have intentionally reduced the resolution of the heat maps where appropriate.

## Results

Our collection of multispectral photographs is the first publicly available database that combines spatial and spectral information in the range relevant for insect vision (http://liu.edu/flower). The Flower Reflectance Database (FReD, Arnold et al. 2010) contains single spectrum measurements without spatial information, and while several hyperspectral or multispectral databases exist, either their spectral range does not cover UV (<400 nm) (Brelstaff et al. 1995; Foster et al. 2006) or they are not public (de Ibarra and Vorobyev 2009).

**Fig. 2:**
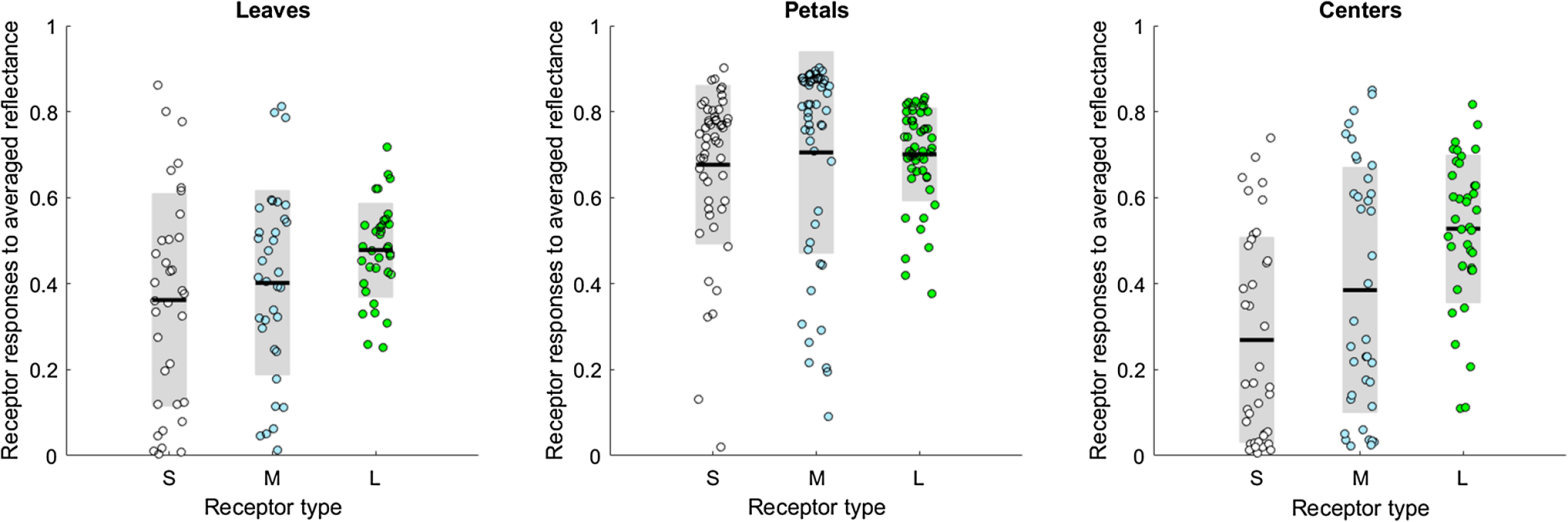
Short-, medium- and long-wavelength receptor responses to the average color of leaves, petals and flower centers. From each of the 53 images in our multispectral database, we selected areas that contained leaves (34 images), petals (52 images) and areas where the nectar and the stamens/carpels can be found (centers, 38 images). From each area, we randomly chose 1000 pixels for estimating the average reflectance spectrum (practically, the color) of each flower part. Finally, we estimated the photoreceptor responses of the bee eye to each leaf, petal and center of each flower species. The *dots* represent the individual receptor responses, the *black lines* indicate their means and the *gray areas* depict the standard deviations. For all areas, the responses of the long-wavelength receptors are the largest and they have the smallest standard deviations (Mean ± SD, leaves: S = 0.36 ± 0.25, M = 0.40 ± 0.21, L = 0.48 ± 0.11; petals: S = 0.68 ± 0.18, M = 0.68 ± 0.23, L = 0.70 ± 0.11; centers: S = 0.27 ± 0.24, M = 0.39 ± 0.28, L = 0.53 ± 0.17)

We examined the photoreceptor responses of the bee eye to the average reflectance spectrum of each leaf, petal and center of each flower species (Fig. 2). For leaves and centers, the responses of the long-wavelength receptors are the largest, and for all areas they have the smallest standard deviations. The low standard deviation means that long-wavelength receptor responses are the most consistent across flower species; thus, long-wavelength receptors are the most useful in informing the bee whether it is approaching a flower or a leaf. The higher standard deviation of the short- and medium-wavelength channels, on the other hand, means that flower colors are spread out in the bee’s visual space. These conclusions are supported by the analysis of the 220 single spectrum measurements downloaded from the Flower Reflectance Database (http://reflectance.co.uk/, Arnold et al. 2010; Supplementary Material, Fig. S5).

**Fig. 3:**
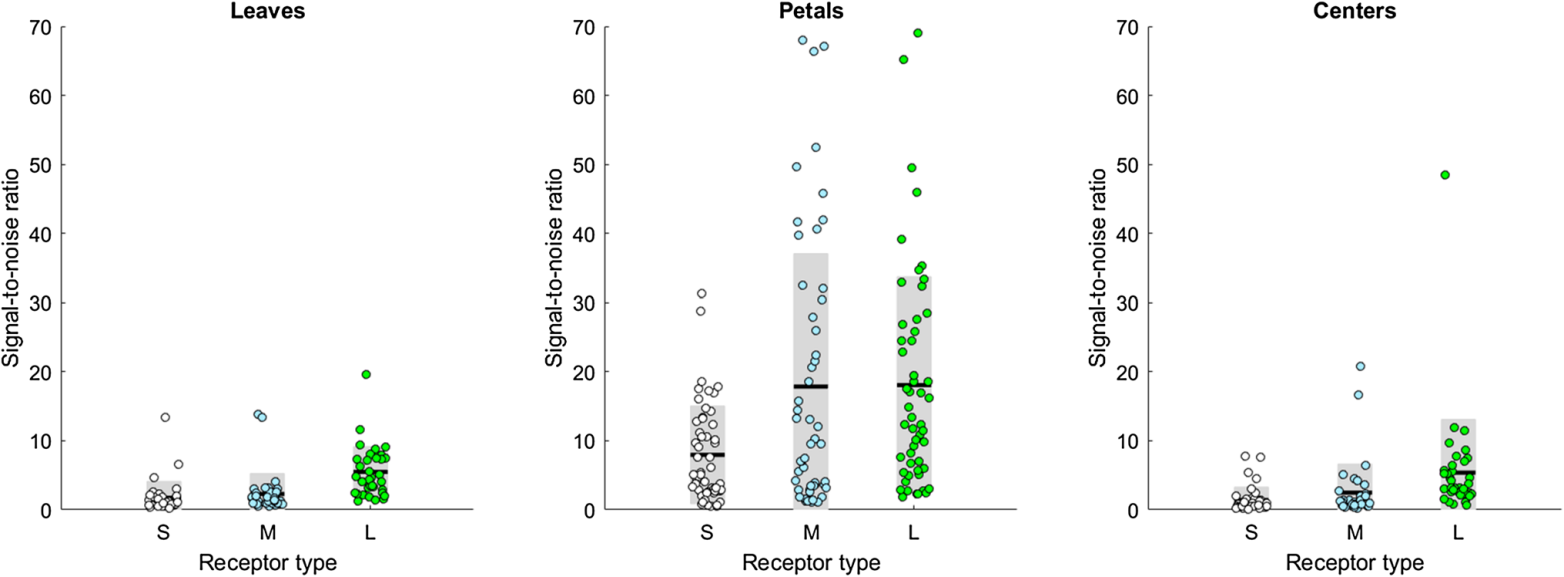
The signal-to-noise ratio is the largest in the long-wavelength channel. From each of the 53 images in our multispectral database, we selected areas that contained leaves (34 images), petals (52 images) and areas where the nectar and the stamens/carpels can be found (centers, 38 images). From each area, we randomly chose 1000 pixels and calculated the photoreceptor responses to the selected pixels. Finally, we calculated the signal-to-noise ratio (SNR) of each area as the mean of receptor responses divided by their standard error. The *dots* represent the individual SNRs of each area of each flower species, the *black lines* indicate their means and the *gray areas* depict the standard deviations. The signal content varies between the different parts of flowering plants—with petals giving nearly an order of magnitude better signal than leaves and centers—but in all cases the long-wavelength receptors provide the most reliable signal with the highest SNR. (Mean ± SD, leaves: S = 1.73 ± 2.40, M = 2.29 ± 2.97, L = 5.49 ± 3.69; petals: S = 7.96 ± 7.09, M = 17.84 ± 19.23, L = 18.08 ± 15.67; centers: S = 1.41 ± 1.87, M = 2.48 ± 4.17, L = 5.39 ± 7.73; Kruskal–Wallis tests *P*_leaves _< 0.001, *P*_petals_ < 0.001, *P*_centers_ < 0.001; Wilcoxon signed-rank tests, all pairwise comparisons *P* < 0.001 except for *P*_petals,M vs L_ = 0.32)

**Fig. 4:**
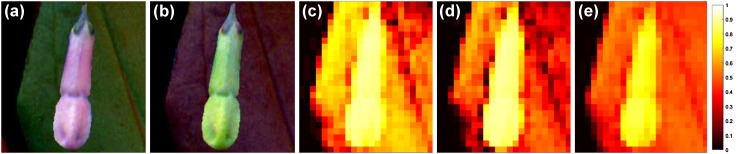
Small spatial variability means homogeneous patches with clear borders. The images show a *Mertensia ciliata* flower (as yet not fully open and in the *pinkish* phase of its life: the floral color changes to *blue* when the flower is fully open) pasted over its leaf. The flower and the leaf were originally next to each other on the same photograph. For more images, see Supplementary Material, Fig. S6. **a** False-colored image for human vision. **b** False-colored image for bee vision. In order to have *colors* visible to humans, we shifted each wavelength higher so that UV becomes *blue*, *blue* becomes *green* and *yellow* becomes *red*. The remaining plots show responses of the short- (**c**), medium- (**d**) and long-wavelength (**e**) photoreceptors. The pictures are intentionally pixelated to mimic the low spatial resolution of the bee eye (total of 10,000 ommatidia, see “Methods”). The long-wavelength channel has the highest signal-to-noise ratio, and this translates into two distinct areas of receptor excitation values

Next, we turned our attention to the spatial structure in the multispectral photos. We calculated the signal-to-noise ratio (SNR) within each leaf, petal or center (Fig. 3). In all cases, the long-wavelength receptors provide the most reliable signal with the highest SNR. When an area has a high signal-to-noise ratio, signals from that area can be easily characterized to be representing the same object. Figure 4 illustrates what small spatial variability and a high signal-to-noise ratio mean for image segregation. Although the average contrast between the *Mertensia ciliata* flower and its leaf is high for the short- and medium-wavelength receptors, the responses themselves are highly variable, too (Fig. 4; see Supplementary Material, Fig. S6 for more images). The small spatial variability and high SNR of the long-wavelength receptors, on the other hand, translate into two distinct areas of receptor response values. Such coding facilitates edge detection and so helps the segregation of the visual field into areas that belong together.

**Fig. 5:**
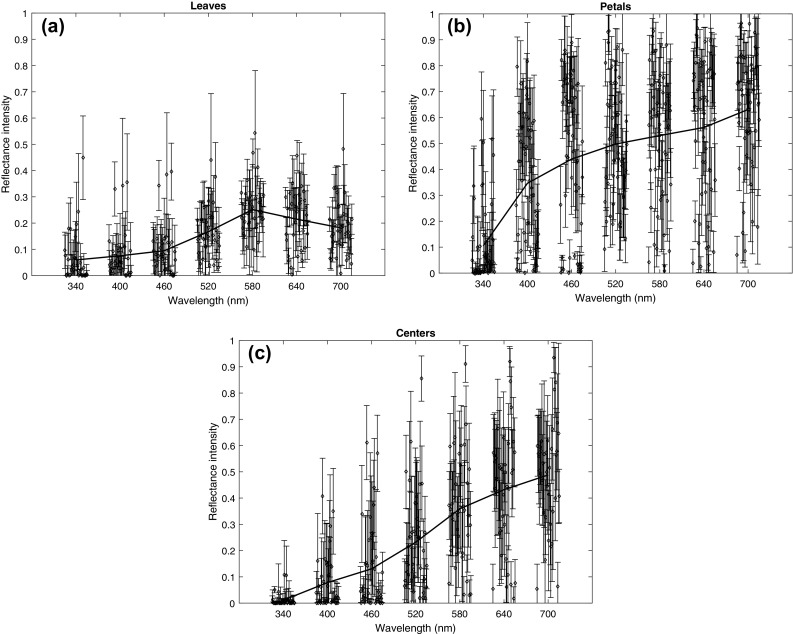
Reflectance properties of leaves, petals and centers. From each of the 53 images in our multispectral database, we selected areas that contained leaves (34 images), petals (52 images) and areas where the nectar and the stamens/carpels can be found (centers, 38 images). From each area, we randomly chose 1000 pixels, and for each of the seven layers, we plotted the mean and standard deviation of their reflectance intensities. The graphs also show the mean of means (*thick line*), i.e., the spectral reflectance function of the ‘average’ leaf (**a**), petal (**b**) or center (**c**). All areas reflect more of long-wavelength light. Standard deviations of reflectance intensities either increase or remain similar, but never decrease with increasing wavelength

**Fig. 6:**
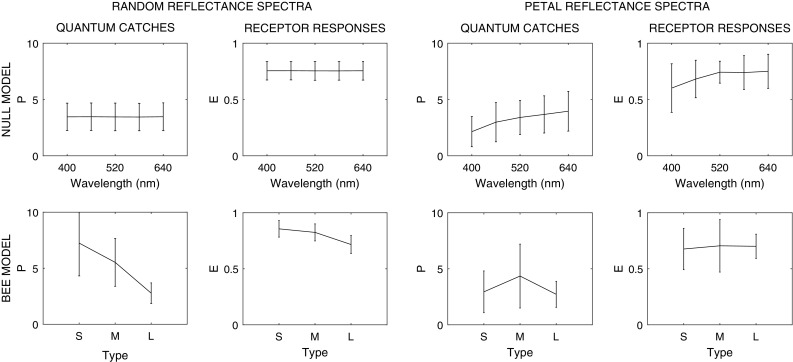
Particulars of the bee eye and flower colors jointly lead to differences in receptor response statistics. The ‘null model’ uses unit background reflectance, unit illumination and unit receptor sensitivities in the calculations (see Supplementary Material, Table S3); the ‘bee model’ uses the receptor sensitivities of bees, the average leaf spectrum as background and the D65 spectrum as illumination. We run both models on 10,000 spectra with randomly generated reflectance intensities (‘random reflectance spectra’) and on the reflectance spectra of the petals from the multispectral photos (‘petal reflectance spectra’) and then plotted the mean and the standard deviations of quantum catches (*P*) and relative receptor responses (*E*). As expected, when the null model is tested on random reflectance spectra, neither quantum catches nor relative receptor responses change alongside the peak sensitivities of the receptors (wavelength). For actual petal *colors*, the null model produces quantum catches and relative receptor responses whose mean increases with increasing peak sensitivity, while the smallest standard deviations appear at peak sensitivity of 520 nm. In the full ‘bee model,’ in response to random reflectance spectra, the mean quantum catches and their standard deviations steeply decrease as the receptors’ peak sensitivity shifts from short- to medium- and then to long-wavelength (type), due to the shape of the receptor sensitivity curves. The nonlinear transformation of *P* to *E*, however, brings the means closer and leads to similar standard deviations. The last two panels show the quantum catches and receptor responses of the bee eye to actual petal colors (note that the receptor responses describe the same data that is plotted on the second panel of Fig. 3). Once all factors are taken into account, their interactions lead to mean relative receptor responses that are equally high for all three receptor types, and to smaller standard deviations in the long-wavelength receptor responses. For more details on parameter tests, see Supplementary Material, Fig. S7

Finally, we investigated the factors leading to the smaller standard deviations and higher SNRs of the long-wavelength receptor responses. Interestingly, it is not the result of flower coloration *per se*: standard deviations of reflectance intensities either increase or remain similar, but never decrease with increasing wavelength (Fig. 5). Parameter tests run on modifications of the null model (one that has random reflectance intensities and unit parameters) point to three different factors that are all essential to generate our results (Fig. 6, Supplementary Material, Fig. S7): (1) the actual receptor sensitivity functions, (2) the actual flower reflectance spectra and (3) the nonlinear transformation of quantum catches to receptor responses. Once all factors are taken into account, their interactions lead to mean relative receptor responses that are equally high for all three types, and to smaller standard deviations in the green receptor responses.

## Discussion

Our investigation into the roles of different spectral channels leads to two main observations. We found that of the three different receptor types, the responses of the long-wavelength receptors (1) are often the largest and they have the smallest standard deviation across a variety of flower species; (2) provide the most reliable signal with the highest signal-to-noise ratio within each individual leaf, petal and flower center. Variation in the photoreceptors’ response and its signal-to-ratio have important implications for subsequent visual processing. Detectability of an object itself is enhanced by (1) high contrast (de Ibarra et al. 2000; Spaethe et al. 2001; for an brief test on how contrast differs among receptor types, see Supplementary Material, Fig. S8), and (2) high variability, as for example produced by iridescence, where the flower appears to be ‘flashing’ as the bee is moving (Whitney et al. 2016). Identifying an object as a flower or a leaf, and identifying groups of pixels as those describing the same object, is a different matter. We suggest that long-wavelength receptors provide the most useful signal for identifying flower petals (as an object class) and for segmenting a visual scene into areas that belong together. The higher variability of the short- and medium-wavelength receptors, on the other hand, means that flower colors are spread out in the bee’s color space and can carry vital information about the species—and thus the nectar reward—of the flower. Our conclusions are in line with experimental results showing the importance of the long-wavelength channel in target detection at the limit of the honeybee’s eye’s resolution (Giurfa et al. 1996; Dyer et al. 2008) and in edge detection (Giger and Srinivasan 1996). Interestingly, medium-wavelength-sensitive receptors yield high signal-to-noise ratios as well, but only for petals (Fig. 3), which might be linked to the supplementary role of medium-wavelength receptors in ventral target detection in honeybees (Giurfa et al. 1999).

When comparing different flower parts, it is worth pointing out how average petal colors—in accordance with their role in advertising the flower for the bee—elicit the highest responses in the receptors. Centers and leaves, on the other hand, are darker and disproportionally so for the UV and blue receptors (Fig. 2). Peripheral signals from blossoms typically include shorter wavelengths than do nectar guides or the colors reflected from the central areas (Mulligan and Kevan 1973; Kevan 1983; Dafni and Kevan 1996). This dim center—bright surround pattern is typical in bee-pollinated flowers (de Ibarra and Vorobyev 2009) and has been linked to increased detectability (Dafni and Kevan 1996; de Ibarra et al. 2001). Similarly, as petals typically have large, smooth, homogenous surfaces, the individual petals provide nearly an order of magnitude better signal than leaves and centers (Fig. 3), thus making petals easier to detect from a distance. In contrast, flower centers are expected to guide the behavior only when the bee is very close (Daumer 1958; Kevan 1983; Mulligan and Kevan 1973; Dafni and Kevan 1996). This contributes to a plant–pollinator system that is highly efficient in terms of visual processing.

The low variability and high signal-to-noise ratio of long-wavelength receptors are the result of the larger range of long-wavelength receptor sensitivity, the fact that petals reflect more light as wavelength increases and the nonlinear transformation of quantum catches to receptor responses altogether. In particular, (1) the sensitivity functions of bee receptors have a secondary peak in the UV region that becomes more pronounced as the main peak is shifting toward longer wavelengths, while the main peak itself is becoming wider as well (Peitsch et al. 1992; Supplementary Material, Fig. S1). As common sense suggests, the wider the receptor sensitivity function is, the less variable the receptor responses become. (2) Not only leaves, but petals and flower centers reflect, on average, more of long-wavelength than short-wavelength light (Fig. 5) so, quantum catches from actual flowers increase with wavelength. This appears to be a general characteristic of flowers and not specific to our database (Daumer 1958; Kevan 1972, 1979, 1983; Mulligan and Kevan 1973; Dafni and Kevan 1996). The subsequent (3) nonlinear transformation of quantum catches to receptor responses means that differences in quantum catches are magnified for the low quantum catches of short-wavelength receptors and are scaled down for the high quantum catches long-wavelength receptors. As the sensitivity functions of generic visual pigments get broader as their peak sensitivity shifts toward longer wavelengths (Govardovskii et al. 2000), and nonlinear responses are expected to be found across the animal kingdom, our findings could potentially apply to a wide range of animals whose edge detection uses predominantly long-wavelength receptor input.

Because of the highly mutualistic nature of plant–pollinator interactions, it is likely that flower colors and the bee’s visual processing apparatus have coevolved, resulting in the bees’ ability to efficiently locate and identify flowers (Kevan and Backhaus 1998). In malacostracan crustaceans and insects, the basic architecture of the optic lobes is similar and was likely present in a common ancestor (Osorio and Bacon 1994; Briscoe and Chittka 2001) that lived long before the rise of angiosperm plants. Insects may have become adapted to use their long-wavelength receptors for edge detection and other tasks because those proved to be the most reliable for natural colors. Accordingly, long-wavelength receptors are the most frequent receptor type in bees (Wakakuwa et al. 2005) as well as in mammals (Jacobs 2013). Later on, various reproductive bracts (including sepals and petals in angiosperms) that evolved to contrast with leaves and stems as much as possible in their pollinators’ eyes were more successful (Kevan and Backhaus 1998). Regardless of the evolutionary history, our results highlight an intriguing example of how specific ecological properties can define more downstream visual processes.

## Electronic supplementary material

Below is the link to the electronic supplementary material.

Supplementary material 1 (DOCX 5608 KB)
