# Supplementary material for: Multispectral images of flowers reveal the adaptive significance of using long-wavelength-sensitive receptors for edge detection in bees
Source: J Comp Physiol A Neuroethol Sens Neural Behav Physiol. 2017 Mar 17;203(4):301–11. doi: 10.1007/s00359-017-1156-x (PMC5389994; doi:10.1007/s00359-017-1156-x)
Supplement: Supplementary file 1 — Supplementary material 1 (DOCX 5608 KB) [file 359_2017_1156_MOESM1_ESM.docx]

**Figure S1. The spectral sensitivity functions of the honeybee (*Apis mellifera*) receptors.** S- short-, M- medium-, L- long-wavelength sensitivity receptors. The sensitivity functions are approximately Gaussian, but with a secondary peak in the UV region that becomes more pronounced as the main peak is shifting towards longer wavelengths (Peitsch et al. 1992).

**
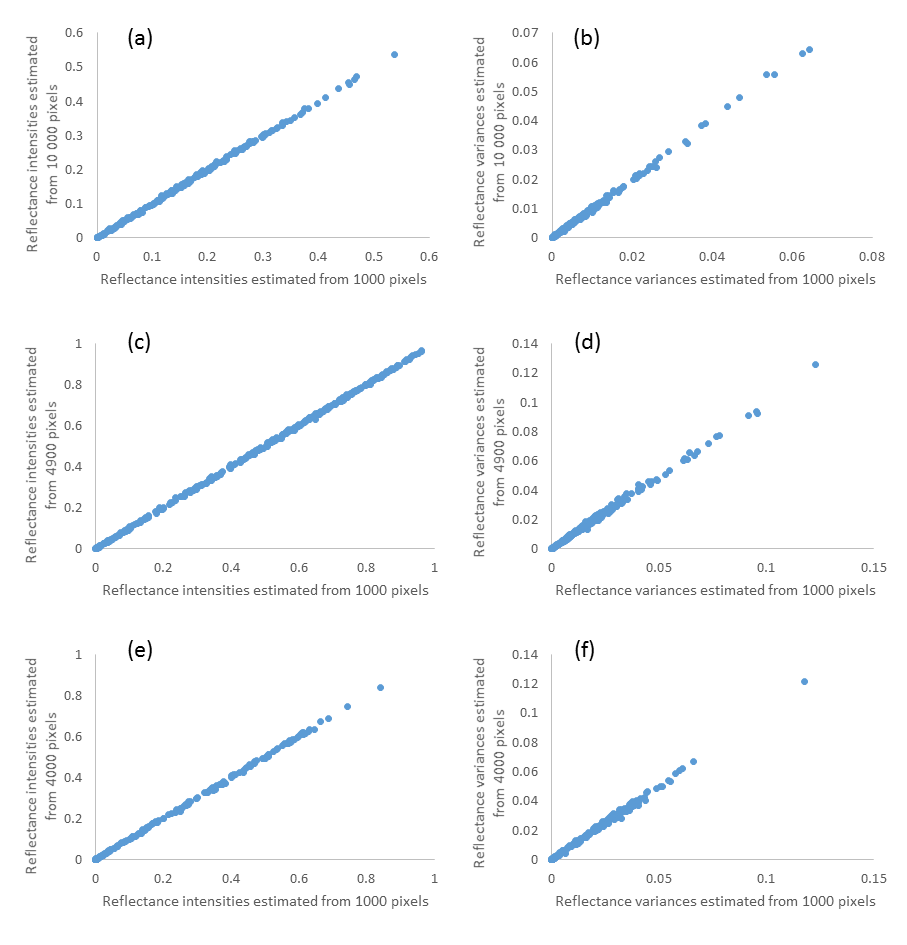
**

**Figure S2. A thousand pixels are sufficient for estimating the spectral reflectance functions.** We plotted the reflectance intensities and their variances for each layer of each photograph estimated from 1000 pixels vs. the maximum number of pixels available. Equally for leaves **(a, b)**, petals **(c, d)** and centers **(e, f)**, the estimations match near perfectly, indicating that the inclusion of more pixels over the 1000 we have used does not carry further information (linear correlation coefficients in all cases are *r* > 0.998).

**Figure S3. Illumination and background reflectance.** We used the standard daylight illumination spectrum (D65; Wyszecki and Stiles 1982), calculated the spectral reflectance function of the ‘average leaf’ from the multispectral photos and adopted another from the flower reflectance database (FReD, Arnold et al. 2010).


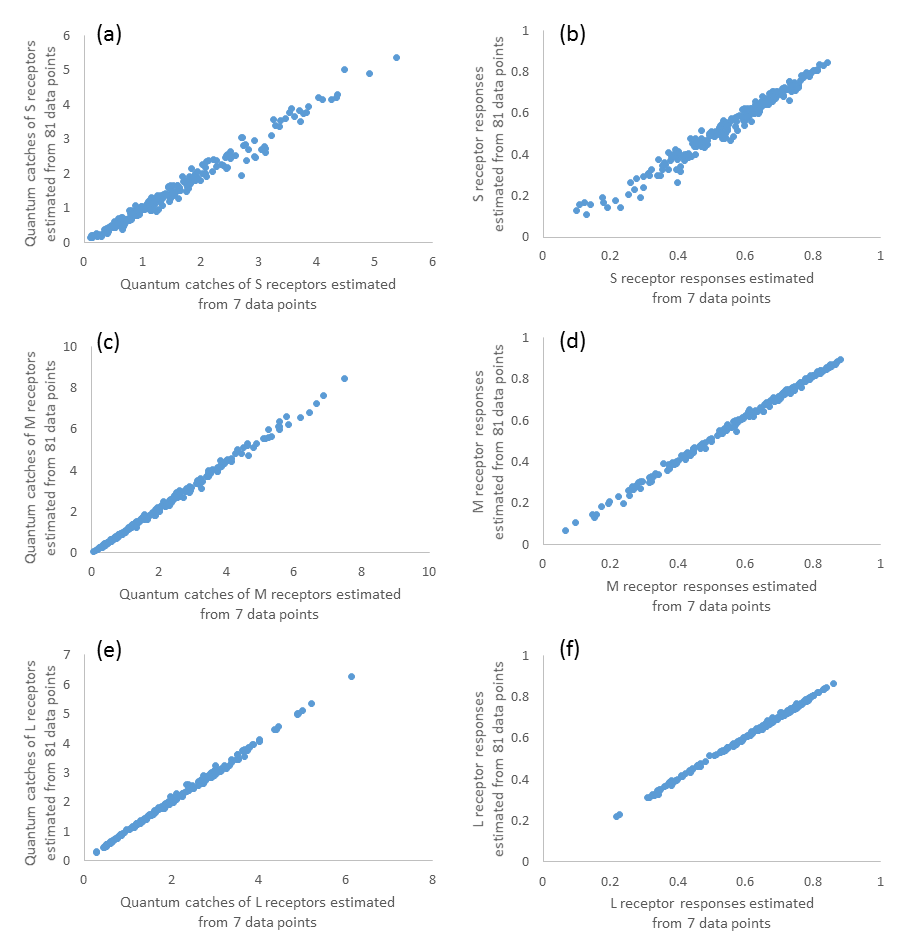


**Figure S4. The low resolution model is sufficient to estimate receptor responses**. As the multispectral photos have only seven layers, we have adjusted the resolution of the model to seven data points per pixel, and the magnitude of noise we have introduced constitutes a valid concern. To address this issue we calculated the quantum catches **(a, c, e)** and the photoreceptor responses **(b, d, f)** of the bee eye for reflectance spectra of 220 flower species downloaded from the open access Flower Reflectance Database (FReD, Arnold et al. 2010), first with a resolution of 7 data points (our model), then with 81 data points (maximum resolution available). The low- and high resolution models show a near perfect match for all three receptor types (linear correlation coefficients for quantum catches: *r_S_*=0.989, *r_M_*=0.998, *r_L_*=0.999, for receptor responses: *r_S_*=0.987, *r_M_*=0.999, *r_L_*=0.999).


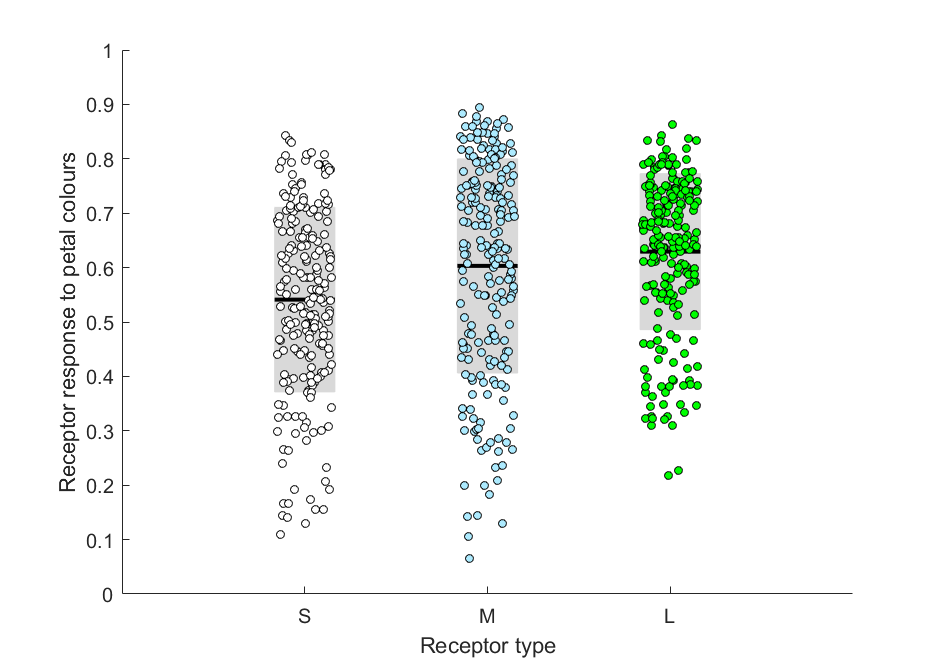


**Figure S5. Single-spectrum measurements support our result that the long-wavelength channel provides the least variable receptor responses across flowers.** We estimated the photoreceptor responses of the bee eye for reflectance spectra of 220 flower species downloaded from the open access Flower Reflectance Database (FReD, Arnold et al. 2010). The long-wavelength receptors provide the least variable, thus most reliable signal for distinguishing flowers from leaves.

1. *Arnica cordifolia*

*(a1) S receptors*


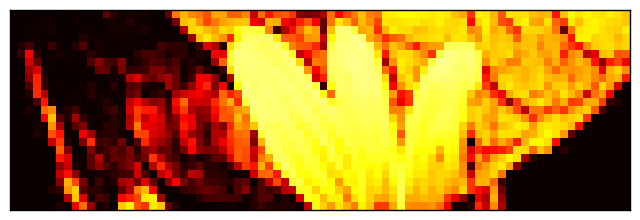

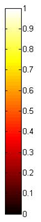


*(a2) M receptors*


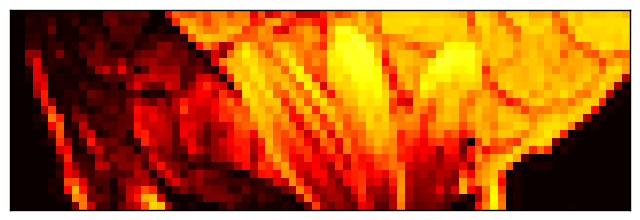

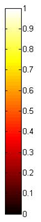


*(a3) L receptors*


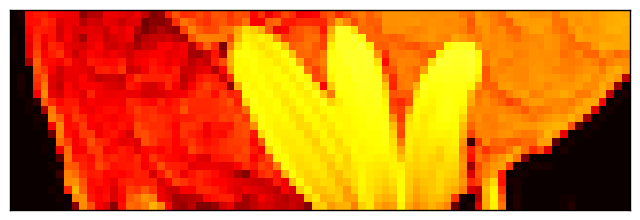

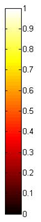


1. *Cardamine pensylvanica*

*(b1) S receptors*


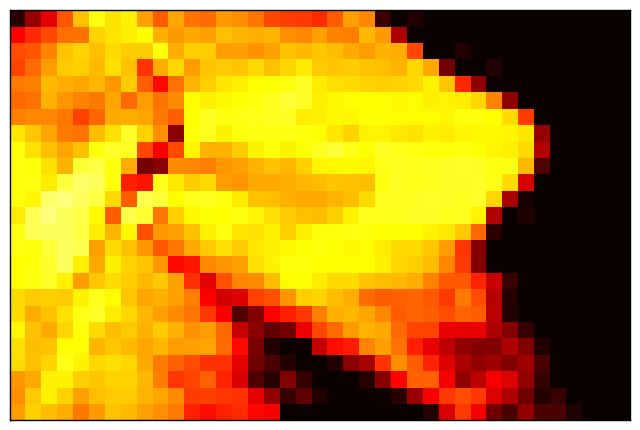

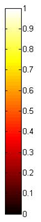


*(b2) M receptors*


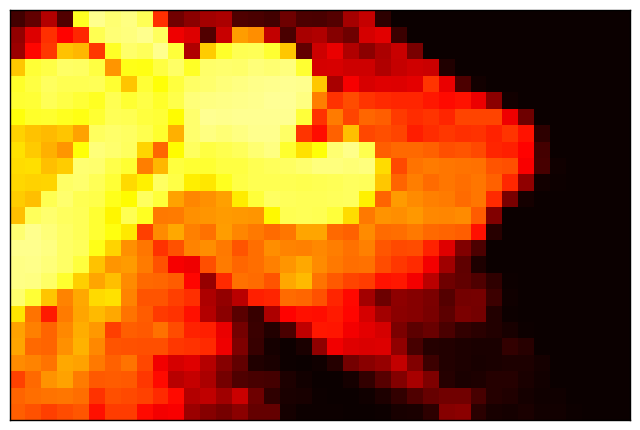

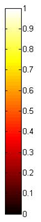


*(b3) L receptors*


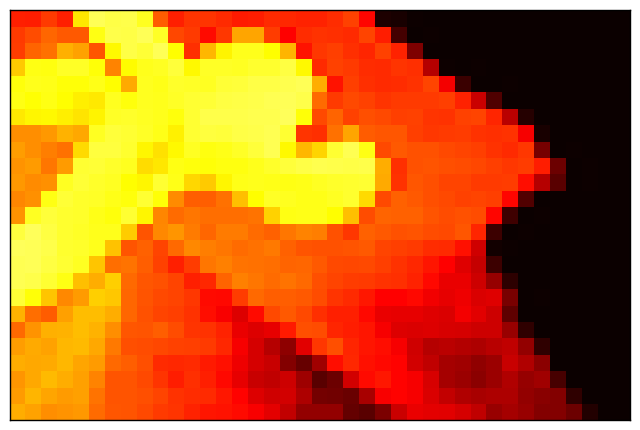

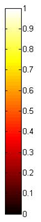


1. *Phlox condensata*

*(c1) S receptors*


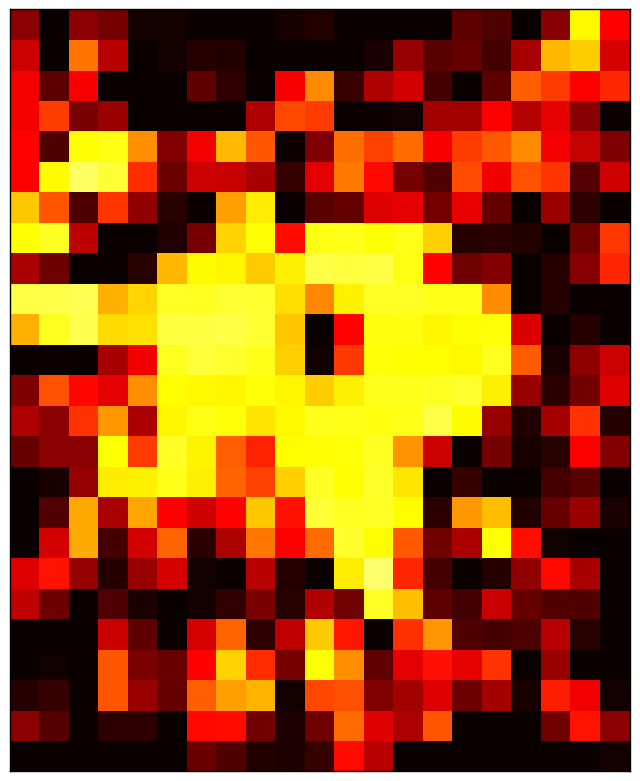

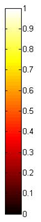


*(c2) M receptors*


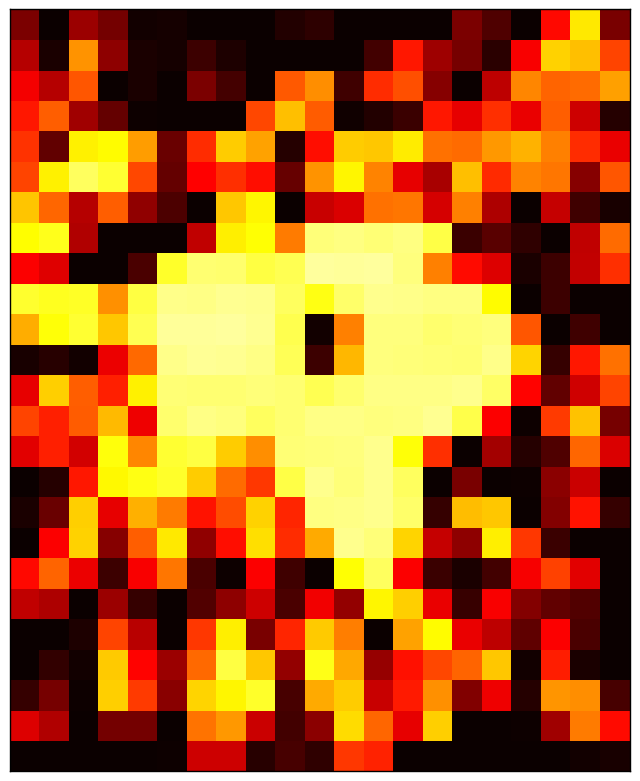

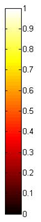


(*c3) L receptors*


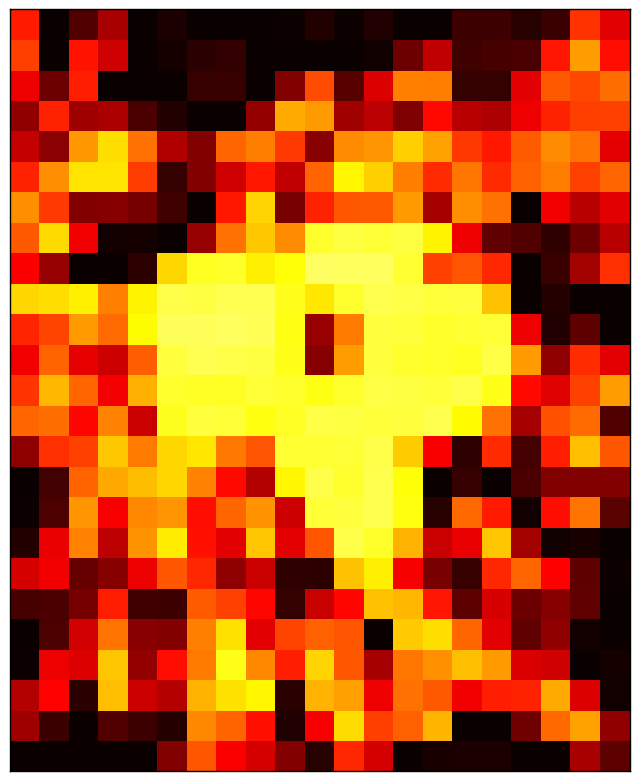

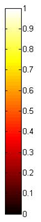


**Figure S6. Further three examples to show the consequences of small spatial variability of receptor response**s. The images show *Arnica cordifolia (a), Cardamine pensylvanica (b)* and *Phlox condensata* *(c)* petals pasted over their own leaves. The plots show photoreceptor responses of the short- **(a1, b1, c1)**, medium- **(a2, b2, c2)** and long-wavelength **(a3, b3, c3)** channels. The pictures are intentionally pixelated to match the low spatial resolution of the bee eye. The green channel has the smallest spatial variability, and this translates into two distinct areas of receptor excitation values **(a, b)**. The picture is less clear in the case of the highly structured *Phlox condensate* leaves, still, the long-wavelength channel gives the clearest signal for identifying leaves **(c)**.


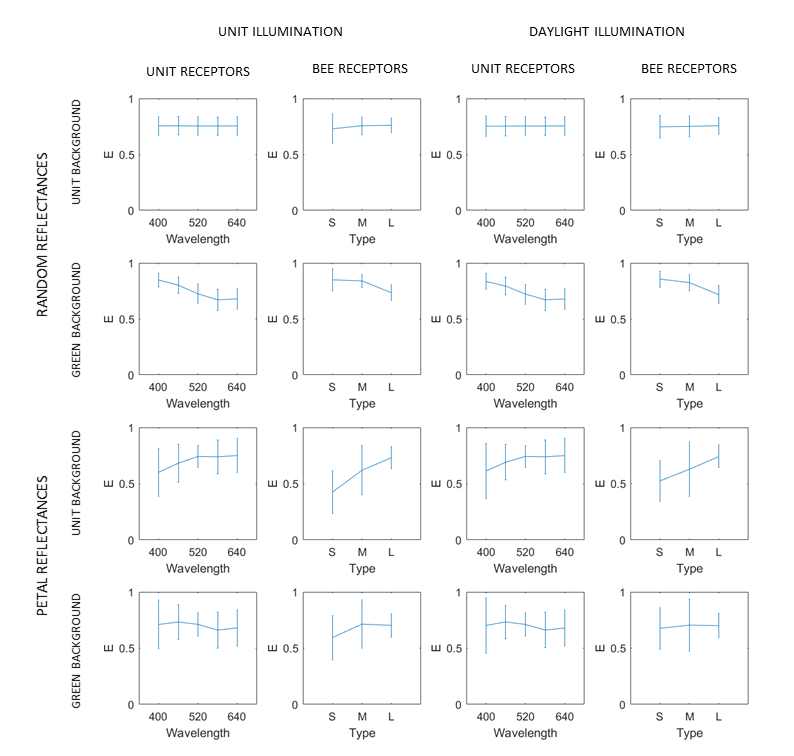


**Figure S7. Parameter tests showing the interactions between the particulars of the bee eye and flower colors.** The plots show the mean and the standard deviations of relative receptor responses (*E*) as a function of the peak receptor sensitivities (*Wavelength*, for the unit receptors) or the receptor type (*Type*, for the bee receptors). For the ‘random reflectances’ rows, we run the model on 10000 spectra with randomly generated reflectance intensities. For the unit background reflectance, unit illumination and unit receptor sensitivities see Supplementary Material, Table S3. Changing the illumination has minor effect on the means and standard deviations of relative receptor responses. Note that illumination has a small and systematic effect on the receptor responses even when the receptors show adaptation (Faruq et al. 2013), and the effect depends on the spectral reflectance function of the object in question (Faruq et al. 2013); however, the small shifts produced by changes in illumination point to different directions and do not lead to a major difference for the means and standard deviations of receptor responses. Changing the color of the background to green lowers the means of receptor responses in the longer wavelength region of the spectrum, but its effect on standard deviations is negligible. The main factors that lead to smaller standard deviations and thus better signal-to-noise ratios for the green region are the actual sensitivity functions of the three types of bee receptors. However, when the receptors adapt to daylight illumination and a green background, the results only hold for actual petal colors and not for random reflectance functions.


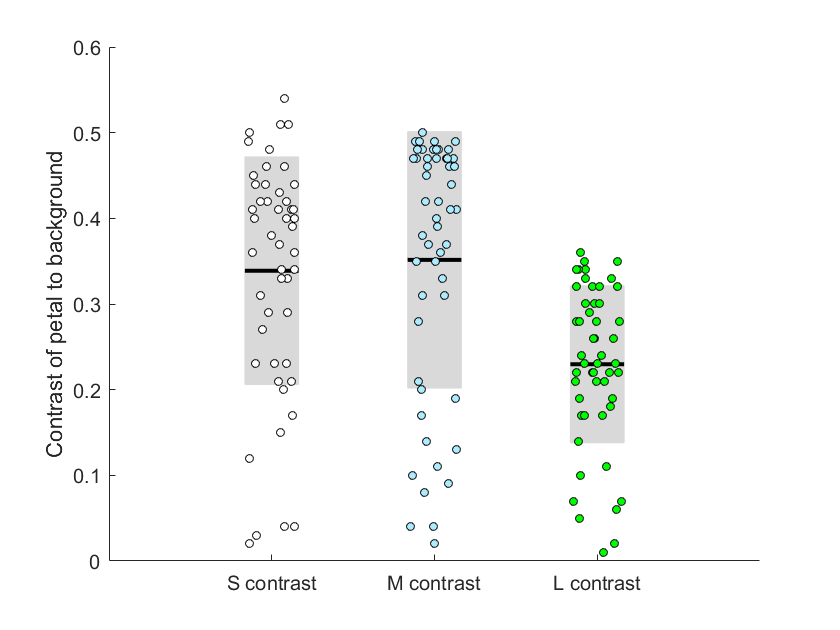

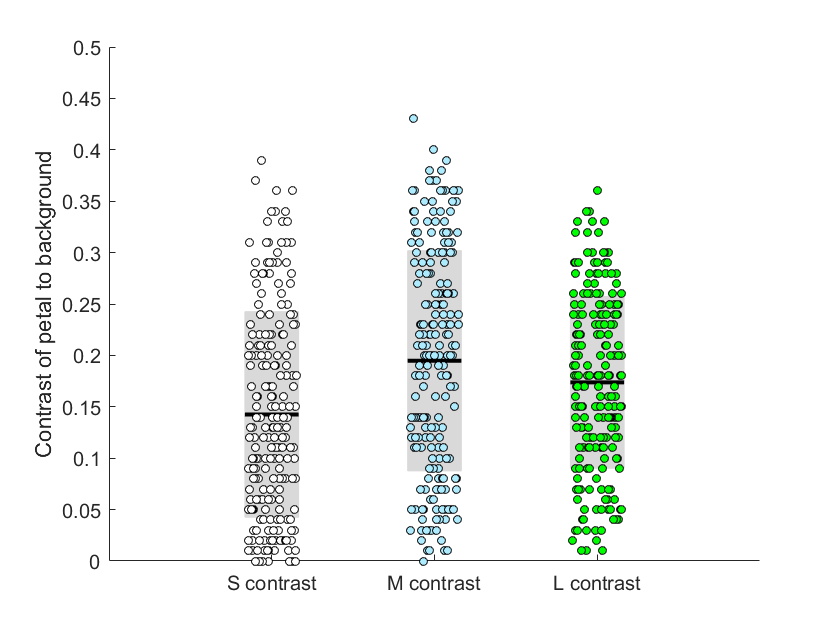


**Figure S8. The long-wavelength channel does not provide the best contrast.** An alternative – an intuitive – explanation for the use of long-wavelength receptors in edge detection would be if they provided the best contrast between different objects. We tested this hypothesis but found no support for it in our analysis. We estimated the photoreceptor responses of the bee eye for the average reflectance spectra calculated from 52 multispectral images of flower petals **(a)** and for reflectance spectrums of 220 flower species downloaded from the open access Flower Reflectance Database (FReD, Arnold et al. 2010) **(b)**. Contrast was defined as the absolute difference between the photoreceptor response to the dominant flower color displayed by the petals and to the leaf background, i.e. it was chosen to measure how much a flower ‘stands out’ from the leaf background in terms of the strength of the neural signal. The dots represent the individual data points, the black lines their means, and the grey areas depict the standard deviations. While the multispectral database and FReD give different estimations on the average contrast, neither of them suggests that the long-wavelength channel should provide better contrast than the shorter wavelength channels. We conclude that good contrast is not the reason behind using long wavelength information for edge detection.

| **Flower name** | **Leaf available** | **Petal available** | **Center available** |
| --- | --- | --- | --- |
| Aconitum columbianum | yes | yes | yes |
| Agoseris glauca | no | yes | yes |
| Arenaria fendleri | no | yes | yes |
| Arnica cordifolia | yes | yes | yes |
| Arnica mollis | no | yes | yes |
| Aster sp. A | yes | yes | yes |
| Aster sp. B | yes | yes | yes |
| Aster foliaceus | yes | yes | yes |
| Cardamine pensylvanica | yes | yes | no |
| Carotium arvensis | yes | yes | yes |
| Castilleja occidentalis | yes | yes | no |
| Claytonia megarhiza | yes | yes | yes |
| Delphinium barbeyi 1 | yes | no | no |
| Delphinium barbeyi 2 | no | yes | yes |
| Erigeron elatior | yes | yes | yes |
| Eritrichium aretioides | no | yes | yes |
| Gentiana calycosa 1 | no | yes | yes |
| Gentiana calycosa 2 | yes | yes | yes |
| Haplopappus parryi | yes | yes | yes |
| Lewisia pygmaea | no | yes | yes |
| Ligularia taraxacoides | no | yes | yes |
| Linum lewisii | no | yes | yes |
| Lomatogonium rotatum | yes | yes | yes |
| Mertensia ciliata blue | yes | yes | no |
| Mertensia ciliata pink | yes | yes | no |
| Moneses uniflora | yes | yes | yes |
| Montia perfoliata | yes | yes | yes |
| Oxytropis splendens 1 | yes | yes | no |
| Oxytropis splendens 2 | no | yes | no |
| Pedicularis groenlandica | no | yes | no |
| Pedicularis racemosa | yes | yes | no |
| Pedicularis sudetica | no | yes | no |
| Penstemon secundiflorus | no | yes | yes |
| Penstemon procerus | yes | yes | yes |
| Pentaphylloides floribunda | no | yes | yes |
| Phacelia sericea | yes | yes | no |
| Phlox condensata | yes | yes | no |
| Primula parryi | yes | yes | yes |
| Primula uniflora | yes | yes | yes |
| Polygonum bistortoides | yes | yes | no |
| Ranunculus adoneus | no | yes | yes |
| Saussaurea alpina | yes | yes | no |
| Saxifraga bronchialis | no | yes | yes |
| Saxifraga hirculus | no | yes | yes |
| Sedium rhodanthemum | yes | yes | yes |
| Senecio atratus | yes | yes | yes |
| Senecio crocatus | yes | yes | yes |
| Senecio triangularis | yes | yes | yes |
| Solidago spathulata | yes | yes | yes |
| Swertia perennis | yes | yes | yes |
| Taraxacum ovinum | yes | yes | yes |
| Trifolium dasyphyllum | yes | yes | no |
| Trifolium parryi | no | yes | yes |

**Table S1. Overview of the data used in the analyses.**

|  | Receptor sensitivity values | | | Illumination | Background reflectance |
| --- | --- | --- | --- | --- | --- |
| wavelength | S | M | L |  |  |
| 340 | 11.571 | 3.576 | 2.303 | 0.176 | 0.048 |
| 400 | 2.002 | 8.428 | 1.962 | 0.520 | 0.058 |
| 460 | 0.208 | 8.332 | 3.960 | 0.909 | 0.085 |
| 520 | 0.012 | 0.433 | 10.248 | 0.972 | 0.136 |
| 580 | 0 | 0 | 8.298 | 0.958 | 0.145 |
| 640 | 0 | 0 | 0.280 | 0.934 | 0.108 |
| 700 | 0 | 0 | 0.073 | 0.890 | 0.112 |

**Table S2. Input parameters used in the simplified model.** We normalized the receptor sensitivity values to have their sum equal the sum of the values of the original 80 data points, and so kept the total sensitivity of the receptors constant.

|  | Unit receptor sensitivity values | | | | | Illumination | Background reflectance |
| --- | --- | --- | --- | --- | --- | --- | --- |
| wavelength | peak 400 | peak 460 | peak 520 | peak 580 | peak 640 |  |  |
| 340 | 0.5 |  | 0 | 0 | 0 | 0.74 | 0.14 |
| 400 | 1 | 0.5 | 0 | 0 | 0 | 0.74 | 0.14 |
| 460 | 0.5 | 1 | 0.5 | 0 | 0 | 0.74 | 0.14 |
| 520 | 0 | 0.5 | 1 | 0.5 | 0 | 0.74 | 0.14 |
| 580 | 0 | 0 | 0.5 | 1 | 0.5 | 0.74 | 0.14 |
| 640 | 0 | 0 | 0 | 0.5 | 1 | 0.74 | 0.14 |
| 700 | 0 | 0 | 0 | 0 | 0.5 | 0.74 | 0.14 |
|  |  |  |  |  |  |  |  |

**Table S3. Input parameters used in the parameter tests in Figure 6.** We chose the average intensity of D65 illumination and the average reflectance of the leaf background as unit illumination and background.

**References**

Arnold SE, Faruq S, Savolainen V, McOwan PW, Chittka L (2010) FReD: the floral reflectance database—a web portal for analyses of flower colour. PLoS ONE 5:e14287

Faruq S, McOwan PW, Chittka L (2013) The biological significance of color constancy: An agent-based model with bees foraging from flowers under varied illumination. J Vis 13:1-14

Peitsch D, Fietz A, Hertel H, Souza J, Ventura DF, Menzel R (1992) The spectral input systems of hymenopteran insects and their receptor-based colour vision. J Comp Physiol A 170:23-40.

Wyszecki G, Stiles WS (1982) Color science, vol 8., Wiley, New York
